# Pilot study on the effectiveness of Reminiscence Therapy on cognition, depressive symptoms, and quality of life in nursing home residents

**DOI:** 10.37825/2239-9747.1018

**Published:** 2020-10-01

**Authors:** I Gil, P Santos-Costa, E Bobrowicz-Campos, A Barata, V Parola, A Coelho, E Santos, ML Almeida, J Apóstolo

**Affiliations:** 1 Department of Elderly Nursing, Nursing School of Coimbra, Coimbra 3046-851, Portugal; 2 The Health Sciences Research Unit: Nursing, Nursing School of Coimbra, Coimbra 3046-851, Portugal; 3 Department of Fundamental Nursing, Nursing School of Coimbra, Coimbra 3046-851, Portugal; 4 Faculty of Psychology and Education Sciences, University of Coimbra, Coimbra 3000-115, Portugal; 5 Rheumatology Department, Centro Hospitalar e Universitário de Coimbra (CHUC), Coimbra 3000-075, Portugal

**Keywords:** aged, reminiscence, cognitive impairment, depression, quality of life

## Abstract

**Aim:**

This study aimed to assess the effectiveness of the group Reminiscence Therapy (RT) on cognition, depressive symptoms, and quality of life (QOL) in older adults recruited in nursing homes.

**Methods:**

A pilot study with a one-group pretest-posttest design was conducted between September 2017 and March 2018 in five nursing homes from central Portugal. A comprehensive RT program (Core program followed by a Follow-up program) was provided to clinically stable volunteers aged 65 years or more, who did not have severe cognitive impairment.

**Results:**

From the 50 older adults (32 women and 18 men, with mean age of 83.32±7.76, and mean education level of 5.48±4.05) considered eligible to participate in the study, 35 (mean age: 84.17±7.46, mean education level of 6.14±4.49) completed the Core Program and 28 completed the Follow-up Program (mean age: 84.25±7.66, mean education level of 6.18±4.57). Based on the Wilcoxon Test, it was observed that the participants’ cognitive performance did not change during the two RT programs. No significant changes were confirmed in relation to depressive symptomatology and QOL.

**Conclusion:**

Although no statistically significant improvements of the older adults’ cognitive function, depressive symptomatology, and quality of life were found, the stabilization of such outcomes are relevant from a clinical viewpoint. Further studies are necessary to confirm these findings.

## I. INTRODUCTION

According to current projections, the old-age dependency ratio in the European Union (EU) is expected to increase from 29.6% in 2016 to 51.2% in 2070 [1]. This implies massive demographical changes, with a significant social and economic impact marked by shifts in the labor market and increased public expenditure.

Not less importantly, rising aging trends are associated with an increment of frailty and morbidity rates, causing dependency on others [2]. This scenario may also sustain the persistent increase of chronic degenerative diseases, such as neurocognitive disorders (NCD). NCDs are described as a syndrome of cognitive impairment that affects cognitive abilities and behavior, thus interfering considerably with a person’s ability to conduct daily life activities [3]. Inherently, complex health status and moderate to high dependency levels result in the need for long-term care [2].

In the EU, a large fraction of long-term care is provided by informal carers, such as family members and friends [1]. However, providing informal care is becoming more challenging than ever. Factors such as the aging trends of the informal carers, the shifts in family structure and roles, and the economic burden on family budgets endanger the concept of home-based long-term care [2].

Complex and demanding scenarios of informal care can lead to a negative impact on the relatives’ health status, reducing their ability to care and to participate in the labor market, as well as trigger feelings of criticism and distantness between the family and the older adult being cared for [4]. This reality often results in the nursing home placement (NHP) of the older adults, depriving them of their social network integrity, environmental landmarks, and higher quality of life [5,6]. In fact, cognitive and functional impairment is the main predictor of the likelihood of NHP due to lack of support and assistance in daily living needs [6,7], either provided by an informal carer or by community-based services [8].

However, NHP can lead to a gradual loss of social roles and purposes, and older adults may be severely affected by a set of restrictions that further hinder their learning capacity, self-esteem, and relationship skills [5]. This reality is strongly associated with the escalation of cognitive decline and depressive symptomatology amongst institutionalized older adults, with a decrease in their perceived quality of life.

In these scenarios, evidence shows that traditional pharmacological interventions do not slow or reverse cognitive function outcomes [9]. Therefore, more recent efforts have focused on non-pharmacological interventions [9]. Amongst these interventions, Reminiscence Therapy (RT) has been identified as potentially beneficial for older adults with cognitive impairment [10]. RT consists of the recovery of emotionally significant memories with the help of multisensory stimuli, in an empathic and accepting attitude. Consequently, RT impels older adults to reassess their life courses, strengthening the perception of their own identity [10].

However, the development and validation of RT programs used in international controlled studies are not always well-defined, which can explain the inconsistent results found in several efficacy studies [5]. In this sense, we aim to carry a pilot study to examine the potential effectiveness of group RT on cognition, depressive symptoms, and quality of life in older adults recruited in nursing homes.

## II. METHODOLOGY

### Study Design

A pilot study with a one-group pretest-posttest design was conducted between September 2017 and March 2018. A convenience sample was recruited in five nursing homes from central Portugal.

### Participants

The study sample was composed of nursing home residents aged 65 years or more. The eligibility criteria for this study were: having sufficient visual and auditory skills to participate actively in therapy-related tasks, and the capacity to remain in the therapy sessions lasting approximately one hour, as well as being to consent their participation in the study. Nursing home residents who were clinically unstable, had severe cognitive decline, and were taking cholinesterase inhibitors and/or antipsychotic medication at the time of recruitment were excluded.

### Intervention

All eligible persons were invited to participate in the RT with 14-week duration [11]. The 14-session Core Program was provided during the first seven weeks. The sessions were held biweekly in groups of 6 to 8 participants. After completing the Core Program, the participants were integrated into the Follow-up Program lasting seven weeks. Also, in this case, the sessions were in groups, once a week.

The RT sessions had a 60-minute duration and were structured in three parts. The first 15 minutes were dedicated to the presentation of the session theme. Then, the participants were invited to engage in activities that focused on the given life period (childhood, school time, youth, professional life, and so on.), highlighting relevant personal moments, customs and traditions, or historical events of that time (for more detailed information, see Table 1). They were also encouraged to reflect on specific issues related to the session theme, introduced by the intervention provider. This part lasted 35 minutes and had as main objective to create opportunities for retrieval of autobiographical memories. The last 10 minutes were dedicated for session conclusion and included a moment of relaxation with abdominal breathing techniques.

Both the Core Program and the Follow-up Program were conducted by nurses who were adequately trained in the implementation of non-pharmacological interventions by the research team. Each session was assisted by two intervention providers. One of them assumed the role of leader, organizing the work of the group and maximizing the group’s potential. The other had a role of facilitator, aiding the older persons with difficulties in hearing, seeing, or understanding the instructions given by the leader, and encouraging less active participants to engage in discussions or activities. The professionals responsible for the implementation of the intervention were provided with a structured manual containing information on the fundamental principles of the Reminiscence Therapy, describing in detail all the intervention sessions and presenting examples of topics to be discussed within the session’s main theme. They were also given a device with digital materials to support the development of the activities. Despite the existence of these resources, the intervention providers were capacitated to propose new topics, according to the interest expressed by the participants, and instructed to use multisensory stimuli to widen the cues that facilitate access to autobiographical memories.

The nurses who provided the intervention were members of the nursing homes’ staff. Other nursing homes professionals (psychologists and pedagogues) were also encouraged to be involved in the planning of the RT sessions, facilitating the organization of physical spaces and access to material resources and ensuring the definition of adequate schedules for the activities’ development. These professionals were also invited to take part in social moments to strengthen their relationship with the residents of the institution.

The resources required for each session included a computer and projector to enable the use of digital support material, as well as the objects corresponding to the themes under discussion (old toys, photos, musical instruments, journals, kitchen or garden utensils, vinyl records, and so on.). Some of these objects could be provided by the participants themselves. A detailed list of all necessary resources and the complementary alternatives was included in the manual.

### Instruments

The screening assessment was based on the semi-structured interview and the 6-item Cognitive Impairment Test (6-CIT). The purpose of the interview was to obtain data on the clinical condition and medication taken by the potential participant. The 6-CIT was used to detect severe cognitive decline.

The 6-CIT is a 6-question screening test that assesses time-space orientation, working memory and attention, and verbal memory, and whose scoring system is reversed [12]. The Portuguese version of this test [13,14] revealed a high test-retest reliability (r = .95) and good internal consistency (α = .88). The global cut-off point for cognitive impairment (i.e., not dependent on the formal education level), calculated for the Portuguese population [13], is ≥ 10. As conditions of mild to moderate decline were considered as not impeding to participate in this study, only persons with score > 21 were excluded. This score indicates that none of the cognitive abilities assessed by the 6-CIT remains preserved.

The outcomes of interest included cognition, depressive symptomatology, and quality of life and were assessed using the Montreal Cognitive Assessment (MoCA), the Geriatric Depression Scale with 10 items (GDS-10), and the short version of the World Health Organization Quality of Life scale - module for older adults (8-item WHOQOL-OLD), respectively. The assessment process lasted up to 30 minutes.

The MoCA is a 10-minute instrument designed for detection of mild cognitive impairment [15]. It is constituted by 13 tasks assessing visuospatial and executive abilities, short-term verbal memory, language, attention, and working memory, and orientation to time and place. The internal consistency of the Portuguese version of this test was revealed to be acceptable (α = .775) [16]. The validation study conducted with Portuguese population has proposed the score below 22 as a threshold for mild cognitive impairment and score below 17 as a threshold for Alzheimer’s disease [17].

The GDS-10, the brief version of the 30-item GDS [18], is used to assess the presence of depressive symptoms in older adults. The respondents are asked 10 questions to which they have to answer yes (if the symptom is present) or no (in the absence of the symptom). The timeframe considered for the assessment process is two weeks. The Portuguese version of the GDS-10 obtained the Cronbach’s alpha value of .841 [19]. The optimal cut-off point for depression screening, indicated for the Portuguese population, is ≥ 2 [20].

The 8-item WHOQOL-OLD is a brief version of the WHOQOL-OLD [21], designed for the comprehensive evaluation of the quality of life in older adults. The WHOQOL-OLD consists of six facets, including Sensory abilities, Autonomy, Past, present, and future activities, Social participation, Death and dying, and Intimacy, that are categorized with a five-point Likert scale. In the European Portuguese version of the instrument [22], the new facet Family/Family Life was included. The 8-item WHOQOL-OLD was developed for use in geriatric care facilities [23]. It is composed of the most representative items of all seven facets of the WHOQOL-OLD and contains additionally one general item on the overall quality of life. The analyses based on the Item Response Theory have shown that none of the items of this brief version is a source of the moderate or severe misfit, or presents a differential functioning associated with age, gender, education level, cognitive status, or depression [23].

Data on outcomes of interest were collected three times, before the implementation of the Core Program (the baseline assessment), after the conclusion of the Core Program and before the beginning of the Follow-up Program (the intermediate assessment), and at the end of the Follow-up Program (the final assessment).

### Procedures

After taking notice of the research, the nursing homes’ team members identified the potential participants and informed them about the study. The older adults who showed availability to integrate the study were contacted personally by the researchers. At this time, the potential participants were given information about the study’s objectives and procedures. All persons who decided to integrate the study were asked to provide written informed consent and then were referred for a screening assessment.

After verifying the inclusion and exclusion criteria, the baseline assessment was conducted. Then, the RT composed of the Core Program and the Follow-up Program was implemented. The intermediate assessment was carried out at the end of the Core Program, and the final assessment after the conclusion of the Follow-up Program.

### Ethical considerations

The study complied with the principles of the Declaration of Helsinki and its recommendations and the protocol was approved by the Ethics Committee of the Health and Sciences Research Unit: Nursing (UICISA:E) (Project identification P406-3/2017). Each participating institution gave formal authorization for the development of the study. Moreover, the plan of the RT sessions considered the weekly activity plan defined for each nursing home, being adapted in case of occurrence of extra activities or unexpected events. All potential participants were informed about the study’s objectives and methods, the potential risks and benefits, and the alternatives to nonparticipation in the study. They were also assured that their participation in the study is voluntary and that anonymity of their identity and confidentiality of data obtained will be preserved. All persons who agreed to participate in the study gave their written and informed consent.

### Data Analysis

Data were analyzed using the Statistical Package for Social Sciences (SPSS), version 24.0. Descriptive statistics were used to characterize the study sample, with measurement data being presented as means ± standard deviation, unless stated otherwise. Because relative changes in outcome variables did not follow a normal distribution, the differences from pre-intervention to post-intervention assessments were tested with non-parametric methods (the Wilcoxon test). The non-parametric Mann-Whitney and Chi-squared tests were also used to carry out the subgroup comparisons. The effect size was estimated according to the following formula: r = Z / √N [24], with r ≥ |.2| and < |.5| representing small effect size, r ≥ |.5| and < |.8| representing medium effect size, and r ≥ |.8| representing large effect size [25].

The associations between continuous variables were examined with Spearman’s correlation coefficient (r). Differences in outcomes of interest were calculated to enable correlation analyses, using the formulas: (i) change after the Core Program = baseline assessment value – intermediate assessment value; and (ii) change after the Follow-up Program = intermediate assessment value - final assessment value. Weak associations were indicated by the coefficients below ±0.4, moderate associations by the coefficients ranging from ±0.4 to ±0.69, and strong associations by the coefficients varying from ±0.7 to ±0.89. The remaining values (equal to or higher than ±0.9) were considered indicators of robust associations [26]. A p-value ≤ .05 was deemed statistically significant.

## III. RESULTS

### Compliance

The flow of participants’ progression during the study is shown in Figure 1. In total, 60 older adults screened for inclusion and exclusion criteria. From these, 50 (32 women and 18 men, with a mean age of 83.32 ± 7.76, and mean education level of 5.48 ± 4.05) were considered eligible for the study, being integrated into the RT. The Core Program was completed by 37 participants. The main reason for dropping out during the Core Program period was the transfer to the other institution (7 individuals). Two older adults withdrew from the study for personal reasons and another one due to health problems. In three cases, the reasons for dropping out remained unknown. The minimum number of sessions was additionally established to ensure the robustness of analyses, and the data obtained with older adults who participated in less than 30% of sessions of the Core Program (i.e., in four sessions or less) were excluded from the statistical treatment. Considering the attendance rates, the final number of participants considered in the analysis was 35. Those 35 participants attended, on average, 11 of the 14 Core Program sessions (10.96 ± 2.96; range: 5–14). Of the 35 older adults who concluded the Core Program and participated in the intermediate assessment, only 30 completed the Follow-up Program. Two did not conclude the Follow-up Program due to institutional transfer, and another three for unknown reasons. Regarding the attendance rates, two participants attended two or fewer sessions (less than 30% of the Follow-up Program sessions), so their data could not be considered in further analyses. Therefore, the impact of the Follow-up Program was examined based on data obtained from 28 participants. On average, those 28 participants were present in five of seven sessions of the Follow-up Program (5.42 ± .96; range: 4–7).

### Characteristics of the participants

Descriptive statistics of older adults who completed the RT programs, being present in at least 30% of program sessions (compliers) and withdrew from the study or did not attend the minimum sessions required are presented in Table 2. Only the period of institutionalization in months presented statistically significant differences between participants who concluded the Core Program and those that withdrew from the program. The use of the Mann-Whitney test showed that the Core Program compliers and dropouts/non-compliers did not differ significantly regarding age, education level, period of institutionalization, the 6-CIT scores, and the scores obtained at baseline in the MoCA, GDS-10, and 8-item WHOQOL-OLD tests (p > .05).

The absence of statistically significant differences was also observed for the compliers and dropouts/non-compliers of the Follow-up Program (p > .05). In this case, the group comparison considered variables of age, education level, period of institutionalization, and the 6-CIT scores, as well as the results of the intermediate assessment obtained in the MoCA, GDS-10, and 8-item WHOQOL-OLD tests.

### Impact of the Reminiscence Therapy Programs on cognition

The Wilcoxon test was used to analyze from baseline to intermediate assessment (Z = −.863, p =.388) and from intermediate to final assessment (Z = .122, p = .903) and showed no significant differences in the MoCA score (for descriptive statistics see Table 3).

The absence of statistically significant changes in the Core Program and the Follow-up Program was also observed for the majority of the MoCA subtests assessing visuospatial and executive abilities (Core Program: Z = 1.928, p =.054; Follow-up Program: Z = −1.537, p =.124), naming (Core Program: Z = 0,500, p = 0,617; Follow-up Program: Z = −0,905, p = 0,336), abstraction (Core Program: Z = −.688, p =.491; Follow-up Program: Z = − .626, p =.531), language (Core Program: Z = −1.219, p =.223; Follow-up Program: Z = −1.428, p =.153), attention and working memory (Core Program: Z = 1.795, p =.073; Follow-up Program: Z = −.744, p =.457), short-term verbal memory (Core Program: Z = −.785, p =.433; Follow-up Program: Z = −2.000, p =.045), and orientation (Core Program: Z = −.234, p =.815; Follow-up Program: Z = − .033, p =.974). However, in case of visuospatial and executive abilities and language, a marginally significant increase after the Core Program was observed. After the Follow-up Program, a statistically significant increase (p = .045) in short-term verbal memory with a small effect size was observed (s = 378).

### Impact of the RT Programs on depressive symptoms and quality of life

Descriptive statistics related to the score obtained in the GDS-10 and the 8-item WHOQOL-OLD tests before and after the Core Program and the Follow-up Program are presented in Table 4.

The variance analysis of the GDS-10 and the 8-item WHOQOL-OLD scores, using the Wilcoxon test, showed no significant changes from the assessment carried out before the Core Program to the assessment conducted after this program’s conclusion (GDS-10: Z = − 1.185, p =.236; 8-item WHOQOL-OLD: Z = −.708, p = .479). No significant pre and post-intervention differences in these two outcomes of interest were found in relation to the Follow-up Program (GDS-10: Z = −.122, p = .903 r = − .01; WHOQOL-OLD: Z = −.885, p = .376).

### Correlations between sociodemographic/clinical variables and changes in outcomes of interest

The study of correlations conducted in relation to the Core Program considered the variables age, education level, and months of institutionalization, as well as changes from baseline to the intermediate assessment on the MoCA, GDS-10, and 8-item WHOQOL-OLD scores. The changes observed in cognitive performance did not correlate significantly with age (r = .293, p = .092), education level (r = .106, p = .550), or period of institutionalization (r = .107, p = .547). The lack of significant associations was also observed for changes in depressive symptomatology and for changes in quality of life in relation to age (r = −.071, p = .689; r = .331, p = .142, respectively), education level (r = −.181, p = .307; r = −.050, p = .778, respectively) and period of institutionalization (r = −.207, p = .240; r = .110, p = .535, respectively).

Regarding the Follow-up Program, the study of correlations focused on the aforementioned sociodemographic/clinical variables and changes from the intermediate assessment to the final assessment on the MoCA, GDS-10, and 8-item WHOQOL-OLD scores. The association observed between age and change in the 8-item WHOQOL-OLD score was shown to be significant and of moderate magnitude (r = −.529, p = .005). However, no significant associations were found between age and change in cognitive performance (r = −.247, p = .213) or age and change in depressive symptomatology (r = .155, p = .445). The education level did not correlate significantly with changes in outcomes of interest (MoCA: r = −.148; GDS-10: r = .297; 8-item WHOQOL-OLD: r = .096). The lack of significant correlations was also verified for changes in outcomes of interest and the period of institutionalization (MoCA: r = −.071, p = .723; GDS-10: r = .210, p = .294; 8-item WHOQOL-OLD: r = −.014, p = .945).

### Correlations between attendance rates and changes in outcomes of interest

The differences from baseline to the intermediate assessment on the MoCA, GDS-10, and 8-item WHOQOL-OLD scores were not associated to the attendance rates registered for the Core Program (MoCA: r = −.060, p = .765; GDS-10: r = .132; p = .511; 8-item WHOQOL-OLD: r = −.157, p = .435). The associations between the Follow-up Program-related changes on the outcomes of interest and attendance rates were also found to be not significant (MoCA: r = .108; p = .585; GDS-10: r = −.361, p = .059; 8-item WHOQOL-OLD: r = −.259, p = .184).

Emphasis should be placed on the statistically negative correlation between the 8-item WHOQOL-OLD and the GDS-10 scores (r = −.785, p = .000).

## IV. DISCUSSION

Considering the aim of this study, and regarding the global effect of the RT program, a stabilization on cognition was observed. Thus, and not confirming statistically significant improvements, the Core Program is potentially beneficial in maintaining cognitive performance. Although the Follow-up Program confirms the cognitive stabilization in general, there is a statistically significant improvement in pre- and post-test scores assessed using the MoCA in the area of deferred evocation (short-term verbal memory). This conclusion may reflect the need to maintain the attendance of the Follow-up Program after the Core Program to ensure the improved performance in this cognitive domain.

It is important to note that, in the studies conducted by Akanuma and associates [27] and by Nakamae and colleagues [28], there were also no improvements in cognitive performance. These authors emphasized that the results may have been influenced by the sample’s reduced dimension and distinct characteristics or, even, by the approach in the implementation, frequency, and duration of the programs, whose sessions were held twice a week in both studies.

It appears to us that, in this study, these facts may also have influenced the results. The sampled population is composed of older adults, with a mean age of 80 years, possessing a low education level. The mean period of institutionalization exceeds two years. Also, the analyzed population presents considerable cognitive decline and depressive symptoms.

Although this study did not find global cognitive improvements associated with the implementation of the RT program, other studies confirmed the benefits of this therapeutic intervention [29,30]. These studies held biweekly sessions and performed the assessment using the Mini-Mental State Examination (MMSE).

Concerning depressive symptoms, no statistically significant changes occurred, so these results may be related to the limitations mentioned above. These findings corroborate the results of found in the study by Akanuma and associates [27], yet, in other authors have identified a statistically significant decrease of depressive symptomatology after the implementation of a RT program [29,31,32].

Regarding the impact of RT on quality of life, several studies confirm an improvement associated with its implementation. However, the impact may be influenced by other factors, as described in the study by Nakatsuka and colleagues [33], in which an increase in this dimension occurs regardless of the implemented non-pharmacological intervention. On the other hand, Siverová & Buzgová state that, despite the positive results observed, it is not possible to ascertain that quality of life is affected only by the RT [34].

In this study, no statistically significant changes associated with the implementation of the RT program were confirmed. However, there is a significant, negative, and moderate correlation between age and changes in quality of life. More specifically, the study revealed that, as people age, their quality of life usually stabilizes, according to the decreased variations in the scores of WHOQOL-OLD 8. From this perspective, RT seemingly contributes, as stated by Pinquart & Forstemeir, to the resolution of conflicts and promotes the sense of identity and self-acceptance, thus allowing older people to “arrange” their lives [35].

RT consists of a person-centered intervention which facilitates communicational processes. However, as stressed by Mileski and associates [36], the implementation of these programs becomes more effective if based on a close interaction between multidisciplinary teams. Specifically, in this study, the articulation between nurses, psychologists, and educators stands out.

The planning of care to people in a cognitive decline process should consider their needs and clinical condition, which may include behavioral and psychological changes, more importantly, depressive symptoms,

In general, institutions in Portugal depend on a low number of nurses in comparison to the high number of residents. This has been described in the literature as a significant barrier to the success of interventions [37]. In turn, teams are frequently composed of very young nurses, and, consequently, throughout this study, some difficulties related to the groups’ dynamics occurred during the sessions. Reinforced training was a solution to attempt to remedy those difficulties.

Among the barriers, described by Mileski and collaborators [36], in the implementation of therapeutic interventions directed at the needs of older adults, the most relevant are difficulties in ensuring team training, quality of the relationship between professionals and residents, and the stage of cognitive decline of the patients.

The insufficient team training was indicated as a barrier in the implementation of the interventions [38], which may entail that institutions adjust their timetables to make these training courses malleable. During this study, in particular, the constant introduction of several nurses in the nursing homes’ teams has led to the scheduling of various workshops to train the facilitators who not always complete them. The moments of training often coincided with when the nurses were being integrated into the institution’s dynamics and, also, in a very initial phase of establishing a therapeutic relationship with the residents.

In what concerns RT, O’Shea and collaborators stress that some residents react negatively to longer programs and some people manifest aggravated depressive symptoms [39]. Projecting images or conversations about past events may not trigger any reaction in people with more advanced dementia but touching gentler and contextualized objects may help to smile and convey a feeling of safety [39,40]. However, facilitators may not always be able to identify the level of compromise precisely and adapt the therapeutic intervention to each patient’s condition. In this sense, audiovisual items were used in implementing this program to trigger memories, but there was also the opportunity to handle old objects, like photographs, pieces of clothing, and so on.

Another aspect to be taken into account in researches on this topic is that RT outcomes may be important to the participants during the session, that is, in real time, and not lead to any permanent therapeutic attitude over time. Thus, temporal improvements do not appear to match improvements in quality of life [37].

### Limitations

Firstly, the loss of a significant number of participants can result in an important limitation of this study, as only 35 of the 50 initially screened older adults carried out the intermediate assessment (at the end of the Core Program), and 28 performed the final assessment (end of the Follow-up Program). Transfer to other institutions was the main cause of the loss of participants.

Secondly, the interpretation of the results is conditioned by the absence of a control group. Thus, it is not possible to determine the natural evolution of the older adults who only benefit from the usual care provided by residential institutions, mainly regarding their cognitive performance, depressive symptoms, and quality of life.

## V. CONCLUSION

Although the results of this study do not confirm a statistically significant improvement of the older adults’ cognitive function, depressive symptomatology, and quality of life, they demonstrate the potential of RT in the stabilization of these outcomes from a clinical point of view. Considering the human development stage and the level of initial cognitive decline of the participants, these results can be understood as a positive therapeutic outcome.

The limitations outlined may have conditioned these results; however, other international studies confirm the therapeutic benefits of RT when implemented in institutional settings. Therefore, given that RT is an easy-to-implement, low-cost intervention that focuses on the person and their individuality, we believe that it should be actively included in the therapeutic plan of older adults with cognitive decline.

Considering the scarcity of research studies in Portugal focused on the effectiveness of structured group RT programs in older adults with cognitive impairment-related outcomes, this pilot study constitutes a starting point for the conduction of further researches with larger samples and more robust designs, such as randomized controlled trials. Further studies should include people of lower ages with early-stage cognitive decline to further explore the therapeutic potential of RT in preventing the progression of cognitive decline and associated symptomatology.

## Figures and Tables

**Table 1:** Themes of the Reminiscence Therapy sessions.

Core Program	Follow-up Program
Presentation of all those involved/background Family First smells/family meals Childhood games, toys, and friends School days Youth Music and songs from another era Professional life/occupation Marriage/partnership and children Gardening and agriculture Means of communication/information Means of transportation, travel, and holidays Holidays and festive seasons Closing session	Ambitions and dreams Days at the beach Fashion Cultural celebrations: Freedom Day Shops and products from another era Anniversaries Closing session

**Table 2:** Characteristics of participants who concluded the RT programs and who dropped out from the study or did not attend the required minimum of programs sessions.

	Core Program		Follow-up Program	
	Participants who concluded the program (n = 35)	Withdrawls and non-compliers (n = 15)	Participants who concluded the program (n = 28)	Withdrawls and non-compliers (n = 7)
**Gender** (male/female)	13/22	5/10	11/17	2/5
**Age** (mean±SD; range)	84.17±7.46; 65–94	81.33±8.32; 67–97	84.25±7.66; 65–94	83.86±7.18; 72–91
**Education level** (mean±SD; range)	6.14±4.49; 0–17	3.69±1.60; 0–6	6.18±4.57; 0–17	6.00±4.47; 4–16
**Marital status** (%)	20% married, 5.7% divorced, 20% single, 54.3% widowed	40% married, 7% single, 53% widowed	21.4% married, 7.1% divorced 14.3% single, 5.1% widowed	14% married, 43% single, 43% widowed
**Institutionalization months completed** (mean±SD; range)	32.26±34.42; 0–142	21.00±3.08; 0–120	21.28±25.73; 0–108	42.00±54.51; 1–142
**6-CIT score** (mean±SD; range)	11.37±6.40; 0–21	9.53±5.58; 0–21	11.86±5.94; 0–21	9.43±0.24; 0–19
**MoCA score** (mean±SD; range)	14.26±5.34; 5–28^a^	13.60±6.31; 3–23^a^	13.50±5.15; 5–28^b^	17.29±5.38; 9–24^b^
**GDS-10 score** (mean±SD; range)	3.03±3.05; 0–10^a^	3.07±2.60; 0–9^a^	2.96±2.99; 0–10^b^	3.29±3.55; 0–9^b^
**8-item WHOQOL-OLD score** (mean±SD; range)	27.63±5.24; 14–37^a^	25.00±4.46; 17–31^a^	2.68±4.38; 19–36^b^	27.43±8.30; 14–37^b^

GDS-10: 10-item Geriatric Depression Scale; MoCA: Montreal Cognitive Assessment; SD: standard deviation; 6-CIT: 6-item Cognitive Impairment Test; 8-item WHOQOL-OLD: short version of the scale World Health Organization Quality of Life - module for older adults.

a score obtained before the implementation of the Core Program (at baseline assessment);

b score obtained before the implementation of the Follow-up Program (at intermediate assessment).

**Table 3:** Changes in cognitive domains over the course of the Reminiscence Therapy programs

	Core Program (n = 35)		Follow-up Program (n = 28)	
	Pre-intervention assessment	Post-intervention assessment	Pre-intervention assessment	Post-intervention assessment
**MoCA total score** (mean±SD; range)	14.26±5.34; 5–28	14.71±6.20; 5–29	13.61±6.06; 5–29	13.61±6.24; 1–29
**Visuospatial and executive abilities**	1.29±0.86; 0–4	1.63±1.50; 0–5	1.39±1.45; 0–5	1.11±1.13; 0–4
**Abstraction**	2.03±0.86; 0–3	2.09±0.92; 0–3	2.07±0.98; 0–3	1.96±1.14; 0–3
**Language**	0.77±0.84; 0–2	0.86±0.81; 0–2	0.71±0.76; 0–2	0.82±0.72; 0–2
**Attention and working memory**	1.86±0.77; 0–3	1.63±0.97; 0–3	1.54±1.00; 0–3	1.32±1.02; 0–3
**Short-term verbal memory**	2.94±1.89; 0–6	3.51±1.77; 0–6	3.50±1.88; 0–6	3.18±1.85; 0–6
**Orientation to time and place**	1.20±1.89; 0–5	1.03±1.62; 0–5	0.68±1.19; 0–5	1.36±1.81; 0–5

MoCA: Montreal Cognitive Assessment; SD: standard deviation.

**Table 4:** Changes in participants’ depressive symptomatology and QoL over the course of the Reminiscence Therapy programs.

	Core Program (n = 35)		Follow-up Program (n = 28)	
	Pre-intervention assessment	Post-intervention assessment	Pre-intervention assessment	Post-intervention assessment
**GDS-10 total score** (mean±SD; range)	3.03±3.05; 0–10	2.80±3.00; 0–10	3.00±3.22; 0–10	3.00±2.80; 0–9
8-item **WHOQOL-OLD tota**l **score** (mean±SD; range)	27.63±5,.4; 14–37	28.26±5.10; 17–39	28.04±5.37; 17–39	28.64±5.14; 16–39

GDS-10: 10-item Geriatric Depression Scale; SD: standard deviation; 8-item WHOQOL-OLD: short version of the scale World Health Organization Quality of Life - module for older adults.
